# Synthesis, *in vitro* inhibitory activity, kinetic study and molecular docking of novel *N*-alkyl–deoxynojirimycin derivatives as potential α-glucosidase inhibitors

**DOI:** 10.1080/14756366.2020.1826941

**Published:** 2020-10-01

**Authors:** Ping Lin, Jia-Cheng Zeng, Ji-Guang Chen, Xu-Liang Nie, En Yuan, Xiao-Qiang Wang, Da-Yong Peng, Zhong-Ping Yin

**Affiliations:** a Jiangxi Key Laboratory of Natural Products and Functional Foods, Jiangxi Agricultural University, Nanchang, China; b College of Science, Jiangxi Agricultural University, Nanchang, China; c College of Pharmacy, Jiangxi University of Traditional Chinese Medicine, Nanchang, China; d State Key Laboratory of Medicinal Chemical Biology and College of Pharmacy, Nankai University, Tianjin, China

**Keywords:** 1-Deoxynojirimycin derivatives, α-glucosidase, inhibitor, structure–activity relationship, docking study

## Abstract

A series of novel *N*-alkyl-1-deoxynojirimycin derivatives **25 ∼ 44** were synthesised and evaluated for their in vitro α-glucosidase inhibitory activity to develop α-glucosidase inhibitors with high activity. All twenty compounds exhibited α-glucosidase inhibitory activity with IC_50_ values ranging from 30.0 ± 0.6 µM to 2000 µM as compared to standard acarbose (IC_50_ = 822.0 ± 1.5 µM). The most active compound **43** was ∼27-fold more active than acarbose. Kinetic study revealed that compounds **43**, **40**, and **34** were all competitive inhibitors on α-glucosidase with ***K_i_***of 10 µM, 52 µM, and 150 µM, respectively. Molecular docking demonstrated that the high active inhibitors interacted with α-glucosidase by four types of interactions, including hydrogen bonds, π–π stacking interactions, hydrophobic interactions, and electrostatic interaction. Among all the interactions, the π–π stacking interaction and hydrogen bond played a significant role in a various range of activities of the compounds.

## 1. Introduction

α-Glucosidase is a type of glucosidases that acts on 1,4-α-bonds, which locate on the brush edge of the small intestine and play a critical role in digestion and absorption of carbohydrates^1^. Inhibition of α-glucosidase is one approach to delay the absorption of glucose and decrease the postprandial blood glucose level^2^. Therefore, α-glucosidase inhibitors are widely used for the prevention and treatment of typeII diabetes millitus^3^. Besides, α-glucosidase participates in other physical and biological processes as well and may also be used as a therapeutic agent for other diseases, such as cancer^4^ and HIV^5^. Today, several types of α-glucosidase inhibitors are being clinically used for the treatment of typeII diabetes millitus, such as acarbose, voglibose, and miglitol^6^. However, these medications also have adverse effects, including abdominal discomfort, diarrhoea, and flatulence^7^. So, developing novel α-glucosidase inhibitors is critical and attractive.

Iminosugars are sugars in which the endocyclic oxygen is replaced by a basic nitrogen atom^8^. As α-glucosidase inhibitor, the best known naturally occurring iminosugar was 1-deoxynojirimycin (1-DNJ), which was first isolated from the roots of mulberry trees^9^. 1-DNJ is currently under clinical evaluation, not only acting as an α-glucosidase inhibitor^10^ but also a potent drug for cancer^11^ and HIV^12^. The accepted mechanism is that 1-DNJ inhibits α-glucosidase by competitively blocking the active site of the enzyme^13^, and the nitrogen atom can mimic the charge of proposed transition states of oxocarbonium ion formed during hydrolysis^14^. Pharmacokinetic studies showed that DNJ & DMJ (structurally related to DNJ) were rapidly eliminated from the body in an intact form by renal excretion, resulting in a weak effect on reducing blood sugar *in vivo* (Nakagawa et al.^15^ and Faber et al.^16^). Modification of 1-DNJ by increasing the alkalinity and introducing hydrophobic groups led to significant changes in the potency and specificity of inhibition. Ardes et al.^17^ and Rawlings et al.^18^ synthesised a number of deoxynojirimycin derivatives by combining different groups with a series of the *N*-alkyl chain length, which showed various degrees of inhibition on α-glucosidase and other enzymes. Zhang et al.^19^ synthesised hybrids of 1-DNJ and quinazoline and obtained fifteen compounds, and some compounds exhibited significant inhibitory activities against the epidermal growth factor receptor (EGFR) tyrosine kinase and α-glucosidase. Some new *N*-alkyl, alkenyl, and benzyl substituted DNJ derivatives incorporating a silicon atom in the substituent were synthesised, which showed activity as potent and selective inhibitors of intestinal glucosidase^20^.

Here, we report for the first time the synthesis of a novel series of 1-DNJ derivatives with benzylidene acetone backbone groups (i.e. cinnamic acid, methyl 4-hydroxycinnamate, Ethyl 4′-hydroxy-3′-methoxycinnamate, and 2′-hydroxychalcone) and different length of alkyl chains, and obtained compounds **25–44**. Moreover, all the compounds were evaluated for their α-glucosidase inhibitory activities. Furthermore, kinetic study and molecular docking were also performed to study the mechanism and discussed the structure–activity relationship.

## 2. Chemistry

**Scheme 1.**
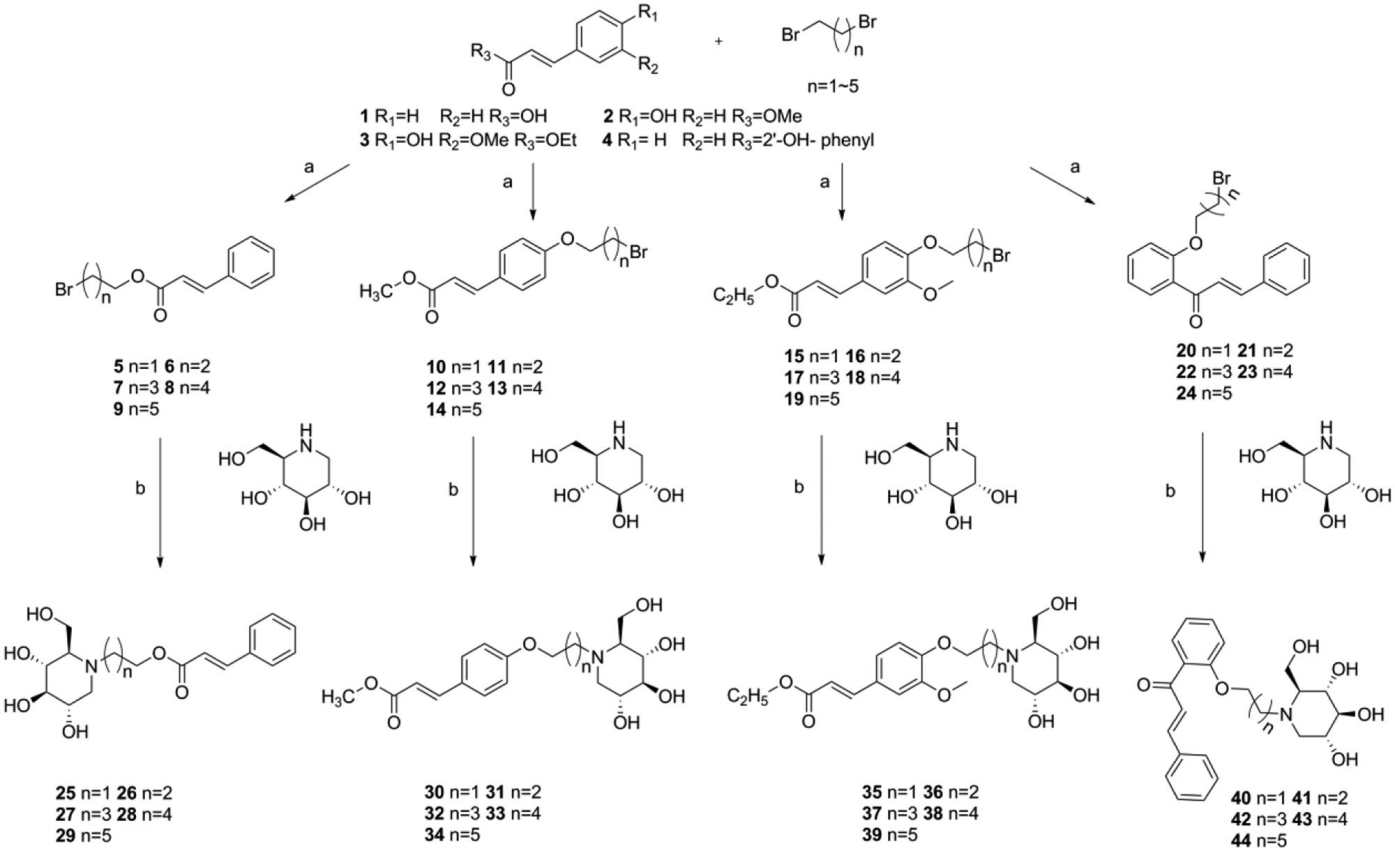
Synthesis of intermediate products and target products of *N*-alkyl-deoxynojirimycin derivatives. Reagents and condition: (a) K_2_CO_3_, acetone, dibromo alkane, 65 °C, overnight or Et_3_N, acetone, dibromo alkane, 65 °C; (b) K_2_CO_3_, DMF, 85 °C, 6 h.

The *N*-alkyl-deoxynojirimycin derivatives **25–44** were synthesised as shown in Scheme 1.

1. To obtain the intermediates **5–24**, the cinnamic acid was reacted with dibromo alkane (i.e. 1, 2-dibromoethane, 1, 3-dibromoproane, 1, 4-dibromobutane, 1, 5-dibromopentane, and 1, 6-dibromohexane) and Et^3^N in acetone at 65 °C, overnight to afford compounds **5–9**. Using the same method above, reactions were also carried out by changing the Et^3^N to K^2^CO^3^ and changing cinnamic acid to methyl 4-hydroxycinnamate, ethyl 4′-hydroxy-3′-methoxycinnamate, 2′-hydroxychalcone to afford compounds **10–14**, **15–19**, **20–24**, respectively.
2. To obtain the target compounds **25–44**, reactions were carried out by dissolving intermediates **5–24**, 1-DNJ and K_2_CO_3_ into DMF, and stirring the mixture at 85 °C for 5–6h. The reaction was monitored by thin-layer chromatography (TLC), and the reaction products were purified with column chromatography using dichloromethane: methane = 25:2 as eluent to afford the pure compounds **25–44**. The structures of all the new synthesised compounds **5–44** were characterised by HRMS, ^1^H and ^13^C NMR spectroscopy.

## 3. Results and discussion

### 3.1. *In vitro* α-glucosidase inhibitory activity

All the synthesised target products **25–44** were screened to check theirs *in vitro* α-glucosidase inhibitory activity. All the synthesised compounds show activity on α-glucosidase with IC_50_ ranging from 30 ± 0.60 µM to 2000 µM as compared with acarbose (IC_50_ = 822.0 ± 1.5 µM). The results were shown in Table 1.

**Table 1.** In vitro α-glucosidase inhibitory activity of compound **25–44**.

Compound	Structure	IC_50_(μM)^a^	Compound	Structure	IC_50_(μM)^a^
**25**		1094.1 ± 1.80	**35**		966.2 ± 0.40
**26**		1099.4 ± 1.10	**36**		>2000
**27**		559.3 ± 0.28	**37**		>2000
**28**		562.1 ± 0.49	**38**		>2000
**29**		976.5 ± 0.70	**39**		>2000
**30**		1272.2 ± 1.20	**40**		160.5 ± 0.60
**31**		>2000	**41**		571.6 ± 0.60
**32**		667.0 ± 1.60	**42**		523.5 ± 0.10
**33**		602.3 ± 1.20	**43**		30.0 ± 0.60
**34**		417.0 ± 0.14	**44**		538.1 ± 0.28
**Acarbose**		822.0 ± 1.50	**1-DNJ**		222.4 ± 0.50

^a^Values are the mean ± SD. All experiments were performed at least three times.

Among all the tested compounds, compound **43** exhibited high α-glucosidase inhibitory activity with IC_50_ of 30.0 ± 0.60 µM which is ∼27-fold higher than acarbose. Similarly, compound **40** showed an excellent activity with IC_50_ of 160.5 ± 0.60 µM, around 5-fold better than acarbose. Others also exhibited inhibitory activities.

**Figure 1.**
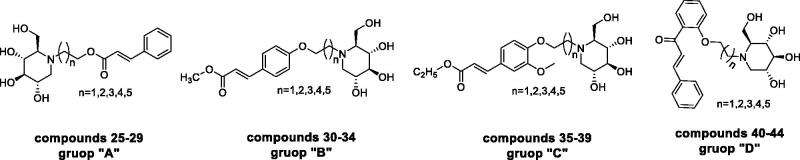
The general structural formula of *N*-alkyl-deoxynojirimycin derivatives.

To better understand the structure–activity relationship, compounds were categorised into four groups “**A**”→“**D**,” cinnamic acid-1-DNJ derivatives **25–29**, methyl 4-hydroxycinnamate –1-DNJ derivatives **30–34**, ethyl 4′-hydroxy-3′-methoxycinnamate-1-DNJ derivatives **35–39**, and 2′-hydroxychalcone-1-DNJ derivatives **40–44** belongs to “**A**,” “**B**,” “**C**,” and “**D**,” respectively. In each group, the difference among those compounds was the length of alkyl chains. The general structural formula was shown in Figure 1

In group “**A**,” compound **27** (purity: 91.5%) with a length of four carbon (*n* = 3) was found to be the most active compound (IC_50_ = 559.3 ± 0.28 µM) as compared to acarbose (IC_50_ = 822.0 ± 1.5 µM), neither increasing nor decreasing the length of alkyl chain would increase the inhibitory activity as observed. When the lengths of alkyl chain were two carbon (*n* = 1, compound **25**, purity: 90.1%) and three carbon (*n* = 2, compound **26**, purity: 93.8%), the inhibitory activities were sharply decreased； and when the lengths were five carbon (*n* = 4, compound **28**, purity: 93.5%) and six carbon (*n* = 5, compound **29**, purity: 93.8%), the inhibitory activities were also decreased. It indicated that the inhibitory activities were highly dependent on the length of the alkyl chain, but without a trend correlation.

In group “**B**,” compound **34** with the length of six carbon (*n* = 5) showed the most inhibitory activity (IC_50_ = 417.0 ± 0.14 µM), and was also the third most active compound among all the synthesised compounds. It is the same as the group “**A**,” the inhibitory activity was poor for the compounds with the alkyl chain containing less than four carbon. While the alkyl chain was long with more than four carbon, the inhibitory activity of the compounds increases as the length of the alkyl chain increases.

In group “**C**,” only compound **35** (purity: 91.2%) showed a low activity with an IC_50_ value of 966.2 ± 0.40 µM. Changing the alkyl chain length of the compounds led to no significant improvement in inhibitory activity. Compared with group “**B**,” it was suggested that the compounds without a methoxy group in the 3′-position of phenyl ring were more active than that with a methoxy group.

In the case of group “**D**,” all compounds were found to have excellent inhibitory activity with IC_50_ values between 30.0 ± 0.60 µM and 571.6 ± 0.60 µM when compared with acarbose (IC_50_=822.0 ± 1.5 µM). Notably, compound **43** with the length of five carbon (*n* = 4) displayed the highest activity with IC_50_ value of 30.0 ± 0.60 µM. This compound was also the most active compound among all the synthesised compounds. In addition, compound **40** with an alkyl chain of two carbon (*n* = 1) was the second most active compound among all the synthesised compounds (IC_50_ = 160.5 ± 0.60 µM). The two compounds were both active than 1-DNJ (IC_50_ = 222.4 ± 0.50 µM) in inhibiting α-glucosidase. Compared to group “**D**” with other groups, compounds in group “**D**” have two phenyl rings, but compounds in other groups have only one, suggesting that the number of phenyl rings plays an important role in compounds’ inhibitory activity.

### 3.2. Kinetic study

**Figure 2.**
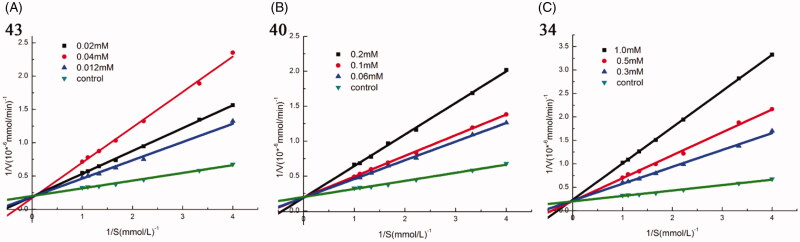
Kinetic analysis of α-glucosidase inhibition by compounds 43, 40, and 34. (A) The Lineweaver–Burk plots in the absence and presence of different concentrations of compound 43; (B) The Lineweaver–Burk plots in the absence and presence of different concentrations of compound 40; (C) The Lineweaver–Burk plots in the absence and presence of different concentrations of compound 34.

To study the inhibition mode of synthesised compounds on α-glucosidase, kinetic studies were performed with the three most active compounds **43**, **40**, and **34**. The type of inhibition and value of ***K_i_*** were determined by Lineweaver–Burk plots. As shown in Figure 2, when increasing concentrations of compound **43**, **40**, and **34**, the ***V_max_*** was not affected, while the ***K_m_*** increased, indicating that all these three compounds were competitive inhibitors for α-glucosidase. The ***K_i_*** values of **43**, **40**, and **34** were 10 µM, 52 µM, and 150 µM, respectively.

### 3.3. Docking study

In order to clarify the interactions between compounds and amino acids in the substrate-binding pocket of α-glucosidase at the molecular level, a molecular docking study was carried out using Autodock Vina^21^. Since the X-ray crystallographic structure of *Saccharomyces cerevisiae* α-glucosidase we used in the experiments has not been reported yet, the 3 D structure of α-glucosidase was conducted with SWISS-MODEL^22^.

Acarbose and the most potent compounds **43**, **40**, and **34** were docked in the active site of the α-glucosidase. In order to explore the structure–activity relationship, compound **41** was also docked. Table 2 showed the results of the molecular docking and detailed interactions, including hydrogen bonds, π–π stacking interactions, hydrophobic interactions, and electrostatic interactions. From the docking study, it was observed that acarbose (Figure 4(A)) interacted with the active site of α-glucosidase via six hydrogen bonds with residues Gln350, Arg312, and Asn241. Additionally, the compound formed several electrostatic interactions with residues Phe157, Phe158, and Phe300.

**Table 2.** The detailed information of molecular docking results of compounds **34**, **40**, **41**, **43**, and acarbose.

Compound	Interactions	Binding Site^a^	Affinity(kcal/mol)
**34**	Hydrogen Bonds	His348, Asp349, Asp214, His279, Glu304, Asn241(3), His239	−8.0
	π–π Interactions	Phe300	
	Hydrophobic Interactions	Phe158, Phe177, Tyr71, Phe300, Leu437	
**40**	Hydrogen Bonds	Glu276(2), Gln350, Asp349, Arg439	−8.7
	π–π Interactions	Phe300, Phe157	
	Hydrophobic Interactions	Ala278, Phe157, Phe158, Phe177, Leu437, Phe311	
	Electrostatic Interaction	Asp349	
**41**	Hydrogen Bonds	Glu276, Gln350, Asp349, Arg439	−8.6
	π–π Interactions	Phe300, Phe157	
	Hydrophobic Interactions	Phe157, Phe177, Phe158, Ala278	
	Electrostatic Interaction	Asp349	
**43**	Hydrogen Bonds	Glu276, Asp349, Arg439, His279, Glu304, Pro309(2), Arg312(2)	−9.2
	π–π Interactions	Phe300, Phe157	
	Hydrophobic Interactions	Leu218, Ala 278, Phe157, Phe300, Phe177, Phe158	
	Electrostatic Interaction	Asp349	
**acarbose**	Hydrogen Bonds	Gln350, Arg312, Asn241(4)	−7.8
	Electrostatic Interaction	Phe157, Phe158, Phe300	

^a^The number in brackets means the number of hydrogen bonds formed with the residues.

**Figure 3.**
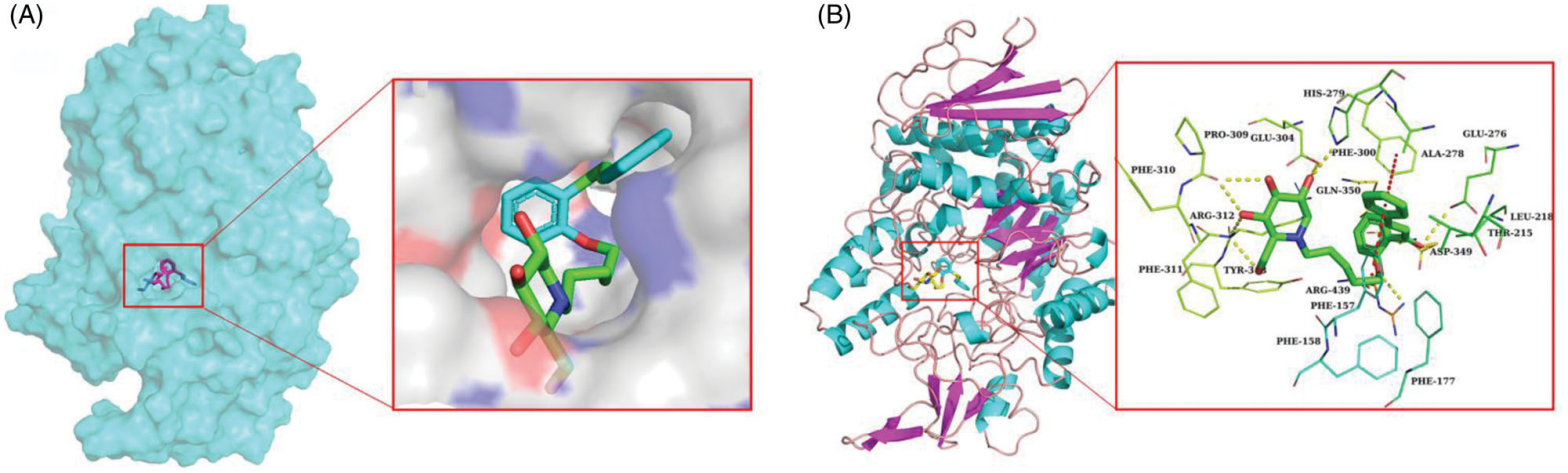
(A) The predicted binding mode of compound **43** in the active site and substrate-binding pocket. (B) the interactions between 43 and modelled α-glucosidase. The yellow dashed lines represent hydrogen bonds and the red dashed lines represented π–π interactions.

The most active compound **43** was well accommodated inside the active site of α-glucosidase (Figure 3(A)) and established nine hydrogen bonds with residues Glu276, Asp349, Arg439, His279, Glu304, Pro309, and Arg312 (Figure 3(B)). Additionally, the phenyl rings of the compound formed two π–π stacking interactions with Phe300 and Phe157. Furthermore, hydrophobic interactions and electrostatic interaction were observed between compound **43** and residues Leu218, Ala 278, Phe177, Phe158, and Asp349. The compound **43** has lower binding energy (−9.2 kcal/mol) than acarbose (−7.8 kcal/mol), suggesting that compound **43** was binding with enzyme more easily and strongly than acarbose.

**Figure 4.**
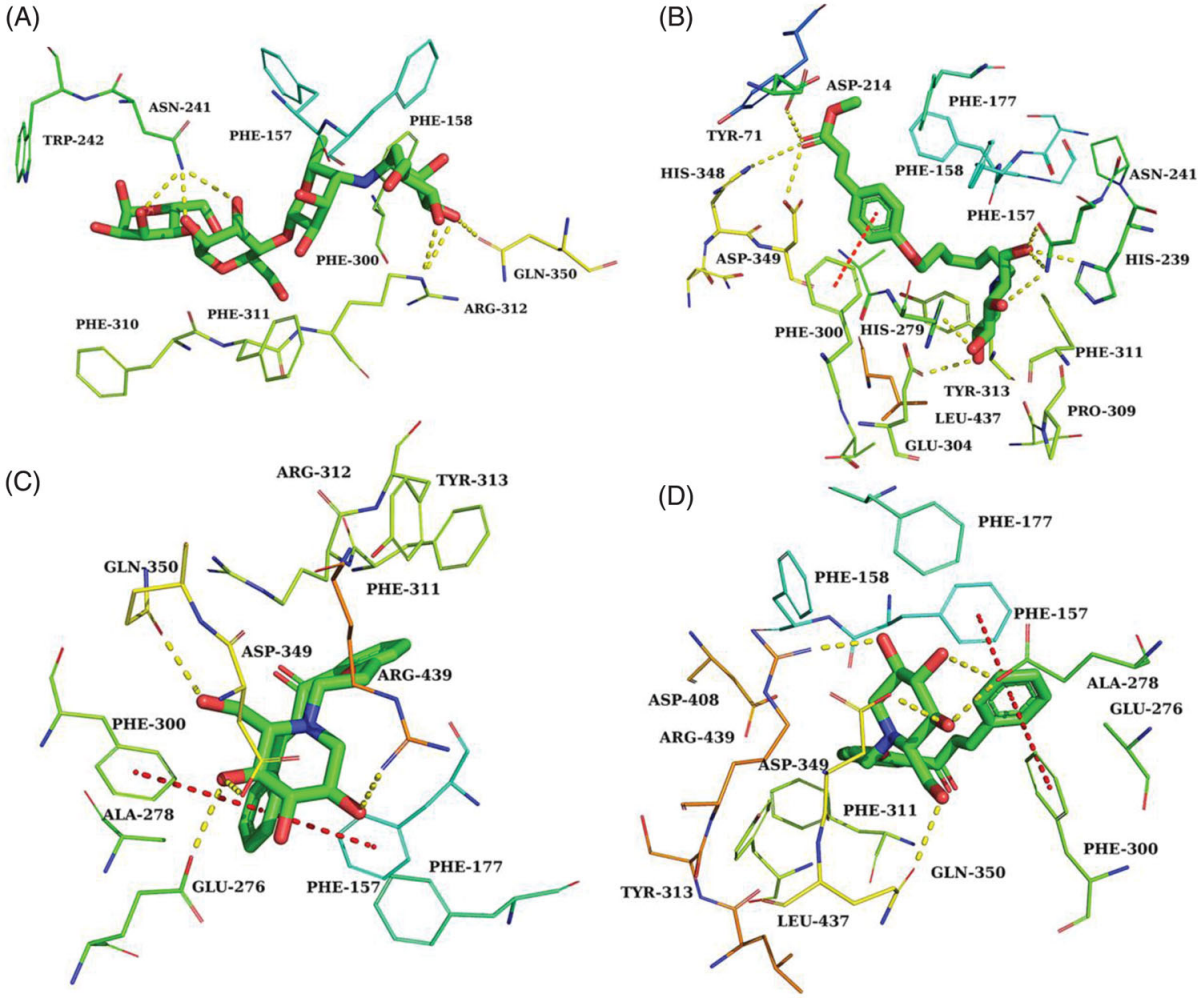
The predicted binding mode of **acarbose** (A), **34** (B), **41** (C) and **40** (D) in the active site pocket. The yellow dashed lines represented hydrogen bonds and the red dashed lines represented π–π interactions.

The second most active compound **40**, similar to compound **43**, formed two π–π stacking interactions with Phe300 and Phe157 (Figure 4(D)). However, compound **40** only formed five hydrogen bonds with residues Glu276, Gln350, Asp349, and Arg439. Furthermore, several hydrophobic interactions and electrostatic interactions were also observed between compound **40** and residues Phe157, Ala278, Phe158, Phe177, Leu437, and Phe311.

In order to explore how the length of the alkyl chain affects the activity on α-glucosidase, compound **41**(*n* = 2), which belongs to the same group as the compounds **43**(*n* = 4) and **40** (*n* = 1), was also docked. As seen in Figure 4(C), except for the number of hydrogen bonds, other interactions were nearly the same as that for compounds **43** and **40**. According to the IC_50_ values, structures, and docking study results, we can infer that the length of the alkyl chain of the compounds affected their α-glucosidase inhibitory activity by affecting the number of hydrogen bonds between the compounds and the enzyme.

In the case of compound **34**, the third most active compound, which formed nine hydrogen bonds with the binding site residues His348, Asp349, Asp214, His279, Glu304, Asn241, His239 and also one π–π interaction with residues Phe157. In addition, several hydrophobic interactions were also observed between molecules and residues Phe158, Phe177, Tyr71, Phe300, and Leu437 (Figure 4(B)). Compared with compound **43**, they both have nine hydrogen bonds, while the most significant difference between them was the number of π–π stacking interactions, the number of π–π stacking interactions for compound **34** was less than that for compound **43**, suggesting that π–π stacking interaction played a significant effect on the inhibitory activity of the compounds.

Studies of the biological activity and molecular docking of these compounds showed that the inhibitory activities were highly dependent on the length of the alkyl chain, but without a trend correlation. Besides, the more phenyl rings in the molecules, the more probabilities to establish π–π stacking interactions between molecules and enzymes, which were responsible for high activity on α-glucosidase. The compounds without a methoxy group in the 3′-position of phenyl ring were more active than that with a methoxy group. The docking results were similar with previous studies. Zeng et al. synthesised a series of *N*-benzyl-deoxynojirimycin derivatives, and the most active compound also established a π–π stacking interactions between molecules and enzymes, which gave the most active compound a strong inhibitory activity to α-glucosidase^23^. Besides, the hydrogen bonds between compounds α-glucosidase and were also played important roles in high activity on α-glucosidase. Shahzad et al. synthesised a series of symmetrical salicylaldehyde-bishydrazine azo molecules. The high activity of compounds is mainly caused by hydrogen bonds^24^, which were the same with us.

## 4. Conclusions

In conclusion, we synthesised a series of novel *N*-alkyl-1-DNJ derivatives **25–44**, all the compounds were tested for their α-glucosidase inhibitory activity. Among them, the compound **43** (IC_50_ = 30.0 ± 0.60 µΜ) was the most active compound, which was ∼27-fold more active than acarbose (IC_50_ = 822.0 ± 1.5 µM) and ∼7-fold more active than 1-DNJ (IC_50_ = 222.4 ± 0.5 µM). The kinetic study revealed that compounds **43**, **40**, and **34** inhibit α-glucosidase via a competitive mechanism with ***K_i_*** of 10 µΜ, 52 µΜ, and 150 µΜ, respectively. The docking study showed that hydrogen bond and π–π stacking interaction played a significant role in the anti-α-glucosidase activity of the synthesised compounds. The numbers of hydrogen bonds and π–π stacking interactions were correlated with and responsible for the compounds’ activities, and the compounds without methoxy group in the 3′-position of phenyl ring were more active than that with a methoxy group.

## 5. Experimental

All starting materials and reagents were purchased from commercial suppliers. α-glucosidase (EC 3.2.1.20) was purchased from Sigma-Aldrich. TLC was performed on Silica gel F-254. Melting points were measured on a microscopic melting point apparatus. The ^1^H NMR and ^13 ^C NMR were measured (DMSO solution) with Bruker spectrometer (500 MHz ^1^H, 125 MHz ^13 ^C). HRMS was performed on AB SCIEX Triple TOF 5600+ with electron spray ionisation (ESI) as the ion source.

### 5.1. General experimental procedure for the syntheses of intermediates 5–9

A solution of cinnamic acid **1** (1 mmol), Et_3_N(3 mmol), dibromo alkane (4 ∼ 5 mmol) in acetone was heated at 65 °C, overnight. After the reaction completed, the mixture was cooled down to room temperature. Water and ethyl acetate were added and extracted three times. The combined organic extracts were dried over Na_2_SO_4_ and then concentrated. Further purification by flash chromatography gave the title compounds.

#### 5.1.1. 2-Bromoethyl cinnamate (5)

Yellow oil; yield: 72%; ^1^H NMR (500 MHz, DMSO) δ 7.77–7.72 (m, 3H), 7.45–7.43 (m, 3H), 6.68 (d, *J* = 16.1 Hz, 1H), 4.50 (t, *J* = 5.6 Hz, 2H), 3.76 (t, *J* = 5.6 Hz, 2H). ^13^C NMR (125 MHz, DMSO) δ 166.31, 145.71, 134.34, 131.11, 129.42, 128.94, 128.78, 117.93, 64.35, 31.27. HRMS (ESI) *m/z* [M + H]^+^: calcd for C_11_H_11_BrO_2_: 255.0015, found 255.0010.

#### 5.1.2. 3-Bromopropyl cinnamate (6)

Yellow oil; yield: 75%; ^1^H NMR (500 MHz, DMSO) δ 7.74–7.69 (m, 3H), 7.45–7.39 (m, 3H), 6.64 (d, *J* = 16.0 Hz, 1H), 4.26 (t, *J* = 6.2 Hz, 2H), 3.63 (t, *J* = 6.6 Hz, 2H), 2.20 (p, *J* = 6.4 Hz, 2H). ^13^C NMR (125 MHz, DMSO) δ 166.57, 145.15, 134.47, 130.95, 129.37, 128.82, 118.32, 62.54, 31.82, 31.46. HRMS (ESI) *m/z* [M + H]^+^: calcd for C_12_H_13_BrO_2_: 269.0172, found 269.0169.

#### 5.1.3. 4-Bromopropyl cinnamate (7)

Yellow oil; yield: 78%; ^1^H NMR (500 MHz, DMSO) δ 7.75–7.70 (m, 2H), 7.66 (dd, *J* = 16.1, 4.7 Hz, 1H), 7.44-7.42 (m, 3H), 6.63 (dd, *J* = 16.0, 3.2 Hz, 1H), 4.18 (dd, *J* = 8.4, 4.5 Hz, 2H), 3.58 (t, *J* = 6.6 Hz, 2H), 1.95–1.88 (m, 2H), 1.82–1.74 (m, 2H).^13^C NMR (125 MHz, DMSO) δ 166.64, 144.95, 134.47, 130.90, 129.36, 128.80, 118.49, 63.68, 35.07, 29.45, 27.49. HRMS (ESI) *m/z* [M + H]^+^: calcd for C_13_H_15_BrO_2_: 283.0328, found 283.0325.

#### 5.1.4. 5-Bromopropyl cinnamate (8)

Yellow oil; yield: 79%; ^1^H NMR (500 MHz, DMSO) δ 7.74–7.69 (m, 2H), 7.66 (d, *J* = 16.1 Hz, 1H), 7.49–7.42 (m, 3H), 6.63 (d, *J* = 16.0 Hz, 1H), 4.16 (t, *J* = 6.5 Hz, 2H), 3.55 (t, *J* = 4.1 Hz, 2H), 1.83 (d, *J* = 7.0 Hz, 2H), 1.71–1.63 (m, 2H), 1.51–1.48 (m, 2H). ^13^C NMR (125 MHz, DMSO) δ 166.66, 144.88, 134.47, 130.90, 129.36, 128.79, 118.55, 64.26, 35.30, 32.32, 27.84, 24.61. HRMS (ESI) *m/z* [M + H]^+^: calcd for C_14_H_17_BrO_2_: 297.0485, found 297.0495.

#### 5.1.5. 6-Bromopropyl cinnamate (9)

Yellow oil; yield: 74%; ^1^H NMR (500 MHz, DMSO) δ 7.76–7.69 (m, 2H), 7.66 (d, *J* = 16.1 Hz, 1H), 7.44–7.42 (m,3H), 6.63 (d, *J* = 16.0 Hz, 1H), 4.14 (t, *J* = 6.6 Hz, 2H), 3.52 (t, *J* = 6.7 Hz, 2H), 1.85–1.77 (m, 2H), 1.69–1.60 (m, 2H), 1.47–1.30 (m, 4H).^13^C NMR (125 MHz, DMSO) δ 166.68, 144.83, 134.49, 130.87, 129.35, 128.77, 118.59, 64.38, 35.45, 32.61, 28.54, 27.68, 25.06. HRMS (ESI) *m/z* [M + H]^+^: calcd for C_15_H_19_BrO_2_: 311.0641, found 311.0652.

### 5.2. General experimental procedure for the syntheses of intermediates 10–24

A solution of methyl 4-hydroxycinnamate **2** (1 mmol), K_2_CO_3_(2 mmol), dibromo alkane (4 ∼ 5 mmol) in acetone was heated at 65 °C, overnight. After the reaction completed, the mixture was cooled down to room temperature. Water and ethyl acetate were added and extracted three times. The combined organic extracts were dried over Na_2_SO_4_ and then concentrated. Further purification by flash chromatography gave the compounds **10–14**. Replaced the Methyl 4-hydroxycinnamate with Ethyl 4′-hydroxy-3′-methoxycinnamate and 2′-Hydroxychalcone, other processes were the same, obtained compounds **15–19**, **20–24**, respectively.

#### 5.2.1. Methyl (E)-3-(4-(2-bromoethoxy) phenyl) acrylate (10)

White solid; yield: 65%; m.p. 85–88 °C; ^1^H NMR (500 MHz, DMSO) δ 7.68 (d, *J* = 8.7 Hz, 2H), 7.63 (d, *J* = 16.0 Hz, 1H), 7.01 (d, *J* = 8.7 Hz, 2H), 6.51 (d, *J* = 16.0 Hz, 1H), 4.42–4.33 (m, 2H), 3.85–3.77 (m, 2H), 3.71 (s, 3H). ^13 ^C NMR (125 MHz, DMSO) δ 167.34, 160.24, 144.64, 130.65, 127.57, 115.89, 115.48, 68.35, 51.78, 31.71. HRMS (ESI) *m/z* [M + H]^+^: calcd for C_12_H_13_BrO_3_: 285.0121, found 285.0121.

#### 5.2.2. Methyl (E)-3-(4-(3-bromoethoxy) phenyl) acrylate (11)

White solid; yield: 68%; m.p. 77–79 °C; ^1^H NMR (500 MHz, DMSO) δ 7.68 (d, *J* = 8.6 Hz, 2H), 7.62 (d, *J* = 16.0 Hz, 1H), 7.00 (d, *J* = 8.7 Hz, 2H), 6.49 (d, *J* = 16.0, 1H), 4.14 (t, *J* = 6.0 Hz, 2H), 3.71 (s, 3H), 3.67 (t, *J* = 6.5 Hz, 2H), 2.29–2.22 (m, 2H).^13^C NMR (125 MHz, DMSO) δ 167.36, 160.71, 144.73, 130.63, 127.27, 115.68, 115.35, 65.98, 51.76, 40.53, 32.20, 31.58. HRMS (ESI) *m/z* [M + H]^+^: calcd for C_13_H_15_BrO_3_: 299.0277, found 299.0279.

#### 5.2.3. Methyl (E)-3-(4-(4-bromoethoxy) phenyl) acrylate (12)

White solid; yield: 70%; m.p. 73–75 °C; ^1^H NMR (500 MHz, DMSO) δ 7.67 (d, *J* = 8.7 Hz, 2H), 7.62 (d, *J* = 16.0 Hz, 1H), 6.98 (d, *J* = 8.7 Hz, 2H), 6.49 (d, *J* = 16.0 Hz, 1H), 4.07 (t, *J* = 6.3 Hz, 2H), 3.71 (s, 3H), 3.61 (t, *J* = 6.7 Hz, 2H), 2.02–1.91 (m, 2H), 1.90–1.79 (m, 2H).^13^C NMR (125 MHz, DMSO) δ 167.38, 160.92, 144.79, 130.61, 127.06, 115.53, 115.32, 67.29, 51.75, 35.25, 29.50, 27.76. HRMS (ESI) *m/z* [M + H]^+^: calcd for C_14_H_17_BrO_3_: 313.0434, found 313.0432.

#### 5.2.4. Methyl (E)-3-(4-(5-bromoethoxy) phenyl) acrylate (13)

White solid; yield: 67%; m.p. 89–91 °C; ^1^H NMR (500 MHz, DMSO) δ 7.66 (d, *J* = 8.7 Hz, 2H), 7.61 (d, *J* = 16.0 Hz, 1H), 6.97 (d, *J* = 8.7 Hz, 2H), 6.48 (d, *J* = 16.0 Hz, 1H), 4.03 (t, *J* = 6.4 Hz, 2H), 3.71 (s, 3H), 3.56 (t, *J* = 6.7 Hz, 2H), 1.92–1.82 (m, 2H), 1.80–1.70 (m, 2H), 1.54 (t, *J* = 7.6 Hz, 2H).^13^C NMR (125 MHz, DMSO) δ 167.39, 161.03, 144.80, 130.61, 126.98, 115.48, 115.30, 67.97, 51.75, 35.49, 32.40, 28.16, 24.71. HRMS (ESI) *m/z* [M + H]^+^: calcd for C_15_H_19_BrO_3_: 327.0590, found 327.0588.

#### 5.2.5. Methyl (E)-3-(4-(6-bromoethoxy) phenyl) acrylate (14)

White solid; yield: 67%; m.p(0).76–79 °C; ^1^H NMR (500 MHz, DMSO) δ 7.66 (d, *J* = 8.4 Hz, 2H), 7.61 (d, *J* = 16.0 Hz, 1H), 6.97 (d, *J* = 8.4 Hz, 2H), 6.48 (d, *J* = 16.0 Hz, 1H), 4.02 (t, *J* = 6.3 Hz, 2H), 3.71 (s, 3H), 3.54 (t, *J* = 6.7 Hz, 2H), 1.88–1.78 (m, 2H), 1.77–1.65 (m, 2H), 1.50–1.39 (m, 4H).^13^C NMR (125 MHz, DMSO) δ 167.39, 161.06, 144.81, 130.61, 126.95, 115.46, 115.29, 68.02, 51.75, 35.55, 32.64, 28.87, 27.74, 25.09. HRMS (ESI) *m/z* [M + H]^+^: calcd for C_16_H_21_BrO_3_: 341.0747, found 341.0745.

#### 5.2.6. Ethyl (E)-3-(4-(2-bromoethoxy)-3-methoxyphenyl) acrylate (15)

White solid; yield: 68%; m.p. 83–86 °C; ^1^H NMR (500 MHz, DMSO) δ 7.59 (d, *J* = 16.0 Hz, 1H), 7.39 (d, *J* = 1.8 Hz, 1H), 7.22 (d, *J* = 1.8 Hz, 1H), 7.01 (d, *J* = 8.3 Hz, 1H), 6.57 (d, *J* = 16.0 Hz, 1H), 4.40–4.31 (m, 2H), 4.18 (q, *J* = 7.1 Hz, 2H), 3.83 (s, 3H), 3.82–3.79 (m, 2H), 1.26 (t, *J* = 7.1 Hz, 3H). ^13 ^C NMR (125 MHz, DMSO) δ 166.92, 149.92, 149.69, 144.83, 128.11, 123.17, 116.54, 113.81, 111.51, 69.04, 60.27, 56.24, 31.59, 14.71. HRMS (ESI) *m/z* [M + H]^+^: calcd for C_14_H_17_BrO_4_: 329.0383, found 329.0382.

#### 5.2.7. Ethyl (E)-3-(4-(3-bromopropoxy)-3-methoxyphenyl) acrylate (16)

White solid; yield: 65%; m.p. 67–70 °C; ^1^H NMR (500 MHz, DMSO) δ 7.58 (d, *J* = 15.9 Hz, 1H), 7.37 (d, *J* = 1.7 Hz, 1H), 7.22 (s, 1H), 7.01 (d, *J* = 8.3 Hz, 1H), 6.56 (d, *J* = 15.9 Hz, 1H), 4.18 (q, *J* = 7.1 Hz, 2H), 4.12 (t, *J* = 6.0 Hz, 2H), 3.83 (d, *J* = 3.7 Hz, 3H), 3.66 (t, *J* = 6.5 Hz, 2H), 2.26 (t, *J* = 6.3 Hz, 2H), 1.26 (t, *J* = 7.1 Hz, 3H). ^13 ^C NMR (125 MHz, DMSO) δ 166.94, 150.41, 149.71, 144.92, 127.73, 123.27, 116.33, 113.38, 111.24, 66.61, 60.25, 56.20, 32.32, 31.62, 14.71. HRMS (ESI) *m/z* [M + H]^+^: calcd for C_15_H_19_BrO_4_: 343.0540, found 343.0535.

#### 5.2.8. Ethyl (E)-3-(4-(4-bromopropoxy)-3-methoxyphenyl) acrylate (17)

White solid; yield: 70%; m.p. 88–90 °C; ^1^H NMR (500 MHz, DMSO) δ 7.58 (d, *J* = 15.9 Hz, 1H), 7.36 (d, *J* = 1.5 Hz, 1H), 7.22 (d, *J* = 8.2 Hz, 1H), 6.99 (d, *J* = 8.3 Hz, 1H), 6.55 (d, *J* = 15.9 Hz, 1H), 4.18 (q, *J* = 7.1 Hz, 2H), 4.04 (t, *J* = 6.3 Hz, 2H), 3.82 (d, *J* = 3.5 Hz, 3H), 3.62 (t, *J* = 6.7 Hz, 2H), 2.01–1.93 (m, 2H), 1.85 (dd, *J* = 9.1, 5.5 Hz, 2H), 1.26 (t, *J* = 7.1 Hz, 3H).^13^C NMR (125 MHz, DMSO) δ 166.96, 150.67, 149.62, 144.99, 127.42, 123.30, 116.14, 113.12, 111.14, 67.87, 60.23, 56.18, 35.28, 29.60, 27.81, 14.71. HRMS (ESI) *m/z* [M + H]^+^: calcd for C_16_H_21_BrO_4_: 357.0696, found 357.0691.

#### 5.2.9. Ethyl (E)-3-(4-(5-bromopropoxy)-3-methoxyphenyl) acrylate (18)

White solid; yield: 64%; m.p. 70–73 °C; ^1^H NMR (500 MHz, DMSO) δ 7.58 (d, *J* = 15.9 Hz, 1H), 7.35 (d, *J* = 1.9 Hz, 1H), 7.22 (dd, *J* = 8.3, 1.9 Hz, 1H), 6.98 (d, *J* = 8.3 Hz, 1H), 6.54 (d, *J* = 15.9 Hz, 1H), 4.18 (q, *J* = 7.1 Hz, 2H), 4.00 (t, *J* = 6.4 Hz, 2H), 3.81 (s, 3H), 3.56 (t, *J* = 6.7 Hz, 2H), 1.91–1.84 (m, 2H), 1.79–1.71 (m, 2H), 1.54 (d, *J* = 7.3 Hz, 2H), 1.26 (t, *J* = 7.1 Hz, 3H).^13^C NMR (125 MHz, DMSO) δ 166.97, 150.80, 149.62, 145.01, 127.32, 123.33, 116.07, 113.06, 111.13, 68.54, 60.23, 56.16, 35.50, 32.41, 28.22, 24.79, 14.71. HRMS (ESI) *m/z* [M + H]^+^: calcd for C_17_H_23_BrO_4_: 371.0853, found 371.0840.

#### 5.2.10. Ethyl (E)-3-(4-(6-bromopropoxy)-3-methoxyphenyl) acrylate (19)

Colourless oil; yield: 65%; ^1^H NMR (500 MHz, DMSO) δ 7.58 (d, *J* = 15.9 Hz, 1H), 7.35 (d, *J* = 1.5 Hz, 1H), 7.21 (d, *J* = 8.2 Hz, 1H), 6.97 (d, *J* = 8.3 Hz, 1H), 6.54 (d, *J* = 15.9 Hz, 1H), 4.18 (q, *J* = 7.1 Hz, 2H), 3.99 (t, *J* = 5.8 Hz, 2H), 3.81 (s, 3H), 3.54 (t, *J* = 6.7 Hz, 2H), 1.87–1.78 (m, 2H), 1.77–1.67 (m, 2H), 1.44 (d, *J* = 2.8 Hz, 4H), 1.26 (t, *J* = 7.1 Hz, 3H).^13^C NMR (125 MHz, DMSO) δ 166.97, 150.83, 149.62, 145.02, 127.28, 123.34, 116.05, 113.01, 111.11, 68.55, 60.22, 56.15, 35.54, 32.65, 28.91, 27.73, 25.12, 14.71. HRMS (ESI) *m/z* [M + H]^+^: calcd for C_18_H_25_BrO_4_385.1009, found 385.0996.

#### 5.2.11. (E)-1-(2-(2-bromoethoxy) phenyl)-3-phenylprop-2-en-1-one (20)

Yellow oil; yield: 64%; ^1^H NMR (500 MHz, DMSO) δ 7.77 (d, *J* = 3.6 Hz, 1H), 7.76 (d, *J* = 2.2 Hz, 1H), 7.61 (d, *J* = 3.7 Hz, 2H), 7.58 (dd, *J* = 7.5, 1.8 Hz, 1H), 7.55–7.52 (m, 1H), 7.45–7.42 (m, 3H), 7.19 (d, *J* = 8.4 Hz, 1H), 7.10 (t, *J* = 7.3 Hz, 1H), 4.50–4.45 (m, 2H), 3.83–3.79 (m, 2H).^13^C NMR (125 MHz, DMSO) δ 191.96, 156.95, 142.69, 135.13, 133.78, 130.86, 130.51, 129.39, 129.35, 129.08, 127.43, 121.61, 113.66, 68.95, 31.73. HRMS (ESI) *m/z* [M + H]^+^: calcd for C_17_H_15_BrO_2_: 331.0329, found 331.0330.

#### 5.2.12. (E)-1-(2-(3-bromoethoxy) phenyl)-3-phenylprop-2-en-1-one (21)

Yellow oil; yield: 66%; ^1^H NMR (500 MHz, DMSO) δ 7.74 (d, *J* = 3.7 Hz, 1H), 7.73–7.72 (m, 1H), 7.56–7.51 (m, 3H), 7.46 (s, 1H), 7.45–7.42 (m, 3H), 7.20 (d, *J* = 8.2 Hz, 1H), 7.08 (t, *J* = 7.4 Hz, 1H), 4.20 (t, *J* = 5.8 Hz, 2H), 3.56 (t, *J* = 6.7 Hz, 2H), 2.23 (p, *J* = 6.3 Hz, 2H).^13^C NMR (125 MHz, DMSO) δ 192.46, 157.30, 142.71, 134.96, 133.63, 130.95, 130.17, 129.46, 129.44, 128.92, 127.43, 121.27, 113.49, 66.46, 32.31, 31.47. HRMS (ESI) *m/z* [M + H]^+^: calcd for C_18_H_17_BrO_2_: 345.0485, found 345.0482.

#### 5.2.13. (E)-1-(2-(4-bromoethoxy) phenyl)-3-phenylprop-2-en-1-one (22)

Yellow oil; yield: 70%; ^1^H NMR (500 MHz, DMSO) δ 7.73 (d, *J* = 3.7 Hz, 1H), 7.72 (d, *J* = 2.1 Hz, 1H), 7.54 (d, *J* = 2.6 Hz, 1H), 7.52 (d, *J* = 2.3 Hz, 2H), 7.49 (s, 1H), 7.46–7.43 (m, 3H), 7.19 (d, *J* = 8.1 Hz, 1H), 7.07 (dd, *J* = 10.9, 3.9 Hz, 1H), 4.13 (t, *J* = 6.0 Hz, 2H), 3.42 (t, *J* = 6.5 Hz, 2H), 1.95–1.87 (m, 2H), 1.86–1.79 (m, 2H). ^13 ^C NMR (125 MHz, DMSO) δ 192.42, 157.65, 142.41, 135.06, 133.66, 130.88, 130.17, 129.45, 129.35, 128.89, 127.62, 121.06, 113.52, 67.73, 34.97, 29.50, 27.93. HRMS (ESI) *m/z* [M + H]^+^: calcd for C_19_H_19_BrO_2_: 359.0641, found 359.0640.

#### 5.2.14. (E)-1-(2-(5-bromoethoxy) phenyl)-3-phenylprop-2-en-1-one (23)

Pale Yellow crystals; yield: 69%; m.p. 58–60 °C; ^1^H NMR (500 MHz, DMSO) δ 7.72 (dd, *J* = 3.7, 1.9 Hz, 2H), 7.53 (s, 1H), 7.51 (d, *J* = 4.7 Hz, 2H), 7.49 (s, 1H), 7.44 (dd, *J* = 2.9, 1.9 Hz, 3H), 7.18 (d, *J* = 8.2 Hz, 1H), 7.06 (t, *J* = 7.4 Hz, 1H), 4.10 (dd, *J* = 5.8, 3.6 Hz, 2H), 3.33 (d, *J* = 5.3 Hz, 2H), 1.73–1.69 (m, 4H), 1.49–1.43 (m, 2H).^13^C NMR (125 MHz, DMSO) δ 192.49, 157.79, 142.23, 135.13, 133.70, 130.85, 130.17, 129.42, 129.34, 128.84, 127.76, 121.01, 113.51, 68.42, 35.00, 32.43, 28.39, 24.89.HRMS (ESI) *m/z* [M + H]^+^: calcd for C_20_H_21_BrO_2_: 373.0798, found 373.0796.

#### 5.2.15. (E)-1-(2-(6-bromoethoxy) phenyl)-3-phenylprop-2-en-1-one (24)

Pale yellow crystals; yield: 65%; m.p. 7 4 ∼ 76 °C; ^1^H NMR (500 MHz, DMSO) δ 7.73 (d, *J* = 3.4 Hz, 1H), 7.71 (d, *J* = 2.0 Hz, 1H), 7.53 (s, 1H), 7.51 (d, *J* = 3.5 Hz, 2H), 7.49 (s, 1H), 7.46–7.42 (m, 3H), 7.17 (d, *J* = 8.1 Hz, 1H), 7.05 (t, *J* = 7.4 Hz, 1H), 4.09 (t, *J* = 6.0 Hz, 2H), 3.35 (t, *J* = 6.8 Hz, 2H), 1.73–1.65 (m, 2H), 1.60–1.53 (m, 2H), 1.39–1.33 (m, 2H), −1.301.26 (m, 2H).^13^C NMR (125 MHz, DMSO) δ 192.46, 157.84, 142.21, 135.12, 133.71, 130.87, 130.19, 129.43, 129.31, 128.83, 127.73, 120.98, 113.46, 68.50, 35.29, 32.47, 29.11, 27.77, 25.33.HRMS (ESI) *m/z* [M + Na]^+^: calcd for C_21_H_23_BrO_2_: 409.0774, found 409.0780.

### 5.3. General experimental procedure for the syntheses of target compounds 25–44

A mixture of intermedial compounds **5** (1 mmol) or other intermedial compounds (**6–24**, respectively), 1-DNJ (1 mmol), K_2_CO_3_ (2 mmol) in DMF was stirred at 85 °C for 6 h. After the reaction completed, the mixture was concentrated under reduced pressure at 90 °C. The product was purified by silica gel and thin layer chromatography to give the title compounds.

#### 5.3.1. 2-(3,4,5-Trihydroxy-2-(hydroxymethyl) piperidin-1-yl) ethyl cinnamate (25)

Brown oil; yield: 33; purity: 90.1%. ^1^H NMR (500 MHz, DMSO) δ 7.76–7.70 (m, 2H), 7.67 (s, 1H), 7.46–7.42 (m, 3H), 7.23 (d, *J* = 15.5 Hz, 1H), 5.22 (s, 1H), 5.15–4.83 (m, 3H), 4.72 (s, 2H), 4.45–4.37 (m, 1H), 4.34–4.26 (m, 1H), 4.25–4.19 (m, 1H), 3.82 (d, *J* = 10.7 Hz, 1H), 3.67 (d, *J* = 4.2 Hz, 1H), 3.62 (s, 1H), 3.59–5.53 (m, 1H), 3.20 (s, 1H), 3.13–3.06 (m, 1H), 3.05–2.94 (m, 1H).^13^C NMR (125 MHz, DMSO) δ 166.79, 144.99, 134.57, 130.97, 129.41, 129.25, 128.83, 118.63, 79.57, 71.22, 69.82, 69.22, 65.83, 62.03, 59.86, 50.97.HRMS (ESI) *m/z* [M + H]^+^: calcd for C_17_H_23_NO_6_: 338.1598, found 338.1601.

#### 5.3.2. 3-(3,4,5-Trihydroxy-2-(hydroxymethyl) piperidin-1-yl) ethyl cinnamate (26)

Brown oil; yield: 35%; purity: 93.8%. ^1^H NMR (500 MHz, DMSO) δ 7.76–7.79 (m, 2H), 7.66 (d, *J* = 16.0 Hz, 1H), 7.49–7.38 (m, 3H), 6.62 (d, *J* = 16.0 Hz, 1H), 4.72 (s, 3H), 4.23 (s, 1H), 4.19–4.09 (m, 2H), 3.82–3.72 (m, 1H), 3.61–3.51 (m, 1H), 3.26–3.18 (m, 1H), 3.08–3.00 (m, 1H), 2.98–2.88 (m, 1H), 2.87–2.80 (m, 1H), 2.03–1.92 (m, 2H), 1.84–1.71 (m, 2H), 1.23 (s, 2H).^13^C NMR (125 MHz, DMSO) δ 166.73, 144.87, 134.52, 130.92, 129.40, 128.83, 118.61, 79.59, 71.20, 69.80, 67.35, 63.25, 59.72, 57.35, 49.09, 24.67.HRMS (ESI) *m/z* [M + H]^+^: calcd for C_18_H_25_NO_6_: 352.1755, found 352.1766.

#### 5.3.3. 4-(3,4,5-Trihydroxy-2-(hydroxymethyl) piperidin-1-yl) ethyl cinnamate (27)

Brown oil; yield: 38%; purity: 91.5%. ^1^H NMR (500 MHz, DMSO) δ 7.76–7.70 (m, 2H), 7.65 (d, *J* = 16.0 Hz, 1H), 7.48–7.37 (m, 3H), 6.65 (d, *J* = 16.0 Hz, 1H), 4.58 (s, br, 3H), 4.16 (t, *J* = 6.6 Hz, 2H), 3.80–3.70 (m, 1H), 3.58–3.53 (m, 2H), 3.25–3.18 (m, 1H), 3.07–3.01 (m, 1H), 2.95–2.90 (m, 1H), 2.85–2.78(m, 1H), 2.44–2.34 (m, 1H), 1.98–1.90 (m, 2H), 1.70–1.53 (m, 2H), 1.53–1.42 (m, 2H).^13^C NMR (125 MHz, DMSO) δ 166.75, 144.86, 134.51, 130.91, 129.39, 129.26, 128.84, 118.64, 79.71, 71.29, 69.92, 67.36, 64.56, 59.68, 57.32, 52.05, 26.77, 21.70.HRMS (ESI) *m/z* [M + H]^+^: calcd for C_19_H_27_NO_6_: 388.1731, found 388.1741.

#### 5.3.4. 5-(3,4,5-Trihydroxy-2-(hydroxymethyl) piperidin-1-yl) ethyl cinnamate (28)

Brown oil; yield: 36%; purity: 93.5%. ^1^H NMR (500 MHz, DMSO) δ 7.76–7.70 (m, 2H), 7.66 (d, *J* = 16.0, 1H), 7.46–7.39 (m, 3H), 6.63 (d, *J* = 16.0, 1H), 4.78 (s, br, 3H), 4.29 (s, 1H), 4.15 (t, *J* = 6.6 Hz, 2H), 3.78–3.69 (m, 1H), 3.64–3.55 (m, 1H), 3.30–3.20 (m, 1H), 3.08 (t, *J* = 9.1 Hz, 1H), 2.95 (t, *J* = 8.8 Hz, 1H), 2.89–2.83 (m, 1H), 2.82–2.76 (m, 1H), 2.46 (s, 1H), 2.11–1.99 (m, 2H), 1.75–1.59 (m, 2H), 1.52–1.38 (m, 2H), 1.37–1.25 (m, 2H).^13^C NMR (125 MHz, DMSO) δ 166.75, 144.89, 134.48, 130.92, 129.40, 128.83, 118.60, 79.43, 70.93, 69.58, 67.07, 64.52, 59.18, 57.05, 52.38, 28.62, 24.41, 23.84. HRMS (ESI) *m/z* [M + H]^+^: calcd for C_20_H_29_NO_6_: 402.1887, found 402.1890.

#### 5.3.5. 6-(3,4,5-Trihydroxy-2-(hydroxymethyl) piperidin-1-yl) ethyl cinnamate (29)

Brown oil; yield: 36%; purity: 93.8%. ^1^H NMR (500 MHz, DMSO) δ 7.75–7.69 (m, 2H), 7.65 (d, *J* = 16.0 Hz, 1H), 7.46–7.39 (m, 3H), 6.63 (d, *J* = 16.0, 1H), 4.83 (s, br, 3H), 4.39 (s, 1H), 4.14 (t, *J* = 6.6 Hz, 2H), 3.78–3.69 (m, 1H), 3.66–3.58 (m, 1H), 3.47–3.38 (m, 1H), 3.32–3.23 (m, 1H), 3.16–3.06 (m, 1H), 3.03–2.92 (m, 1H), 2.92–2.85 (m, 1H), 2.86–2.78 (m, 1H), 2.11 (s, 2H), 1.70–1.57 (m, 2H), 1.51–1.41 (m, 2H), 1.39–1.32 (m, 2H), 1.30–1.19 (m, 2H).^13^C NMR (125 MHz, DMSO) δ 166.75, 144.88, 134.48, 130.92, 129.39, 128.82, 118.60, 79.16, 70.60, 69.27, 66.89, 64.48, 58.63, 56.72, 52.43, 28.67, 26.95, 25.77, 24.42.HRMS (ESI) *m/z* [M + Na]^+^: calcd for C_21_H_31_NO_6_: 416.2044, found 416.2061.

#### 5.3.6. Methyl(E)-3-(4-(2-(3,4,5-trihydroxy-2-(hydroxymethyl) piperidin-1-yl) ethoxy) phenyl) acrylate (30)

Pale brown solid; yield: 40%; m.p. 185–187 °C; purity: 90.2%. ^1^H NMR (500 MHz, DMSO) δ 7.66 (d, *J* = 6.5 Hz, 2H), 7.61 (d, *J* = 16, 1H), 6.97 (d, *J* = 8.0 Hz, 2H), 6.48 (d, *J* = 16.0, 1H), 4.71 (s, br, 3H), 4.28 (s, 1H), 4.13 (s, 2H), 3.71 (s, 3H), 3.59 (s, 1H), 3.27–3.13 (m, 2H), 3.03 (s, 1H), 2.95 (s, 2H), 2.86 (s, 1H), 2.18 (s, 1H), 2.09 (s, 2H).^13^C NMR (125 MHz, DMSO) δ 167.42, 161.09, 144.85, 130.62, 126.90, 115.42, 115.32, 79.69, 71.24, 69.89, 68.09, 67.34, 59.55, 57.24, 52.00, 51.56.HRMS (ESI) *m/z* [M + Na]^+^: calcd for C_18_H_25_NO_7_: 390.1523, found 390.1526.

#### 5.3.7. Methyl(E)-3-(4-(3-(3,4,5-trihydroxy-2-(hydroxymethyl)piperidin-1-yl)ethoxy)phenyl)acrylate (31)

Pale brown solid; yield: 38%; m.p. 104–107 °C; purity: 92.2%. ^1^H NMR (500 MHz, DMSO) δ 7.66 (d, *J* = 8.6 Hz, 2H), 7.61 (d, *J* = 16.0 Hz, 1H), 6.97 (d, *J* = 8.6 Hz, 2H), 6.48 (d, *J* = 16.0 Hz, 1H), 4.86 (s, 3H), 4.46–4.15 (m, 1H), 4.05 (d, *J* = 5.6 Hz, 2H), 3.77 (d, *J* = 10.6 Hz, 1H), 3.71 (s, 3H), 3.64 (d, *J* = 22.4 Hz, 1H), 3.00 (t, *J* = 52.0 Hz, 4H), 2.09 (s, 2H), 1.90 (d, *J* = 11.2 Hz, 2H). ^13 ^C NMR (125 MHz, DMSO) δ 167.38, 161.06, 144.81, 130.59, 126.96, 115.48, 115.34, 89.65, 79.65, 71.34, 69.88, 67.31, 66.81, 59.80, 57.52, 51.73, 49.01, 25.11.HRMS (ESI) *m/z* [M + H]^+^: calcd for C_19_H_27_NO_7_: 382.1860, found 382.1859.

#### 5.3.8. Methyl(E)-3-(4-(4-(3,4,5-trihydroxy-2-(hydroxymethyl)piperidin-1-yl)ethoxy)phenyl)acrylate (32)

Pale brown solid; yield: 35%; m.p. 157–160 °C; purity: 95.2%. ^1^H NMR (500 MHz, DMSO) δ 7.65 (d, *J* = 8.7 Hz, 2H), 7.61 (d, *J* = 16.0 Hz, 1H), 6.97 (d, *J* = 8.7 Hz, 2H), 6.47 (d, *J* = 16.0 Hz, 1H), 4.69 (s, br, 3H), 4.25–4.09 (m, 1H), 4.06–4.00 (m, *J* = 6.2, 4.6 Hz, 2H), 3.75 (d, *J* = 11.4 Hz, 1H), 3.71 (s, 3H), 3.61–3.54 (m, 1H), 3.22 (s, 1H), 3.10–3.01 (m, 1H), 2.94 (s, 1H), 2.89–2.78 (m, 2H), 2.41 (s, 1H), 1.94 (s, 2H), 1.76–1.60 (m, 2H), 1.59–1.47 (m, 2H).^13^C NMR (126 MHz, DMSO) δ 167.42, 161.09, 144.85, 130.62, 126.90, 115.42, 115.32, 79.69, 71.24, 69.89, 68.09, 67.34, 59.55, 57.24, 52.00, 51.71, 26.95, 21.64.HRMS (ESI) *m/z* [M + Na]^+^: calcd for C_20_H_29_NO_7_: 418.1836, found 418.1840.

#### 5.3.9. Methyl(E)-3-(4-(5-(3,4,5-trihydroxy-2-(hydroxymethyl)piperidin-1-yl)ethoxy)phenyl)acrylate (33)

Pale brown solid; yield: 37%; m.p. 85–86 °C; purity: 92.1%. ^1^H NMR (500 MHz, DMSO) δ 7.66 (d, *J* = 8.7 Hz, 2H), 7.61 (d, *J* = 16.0 Hz, 1H), 6.96 (d, *J* = 8.2 Hz, 2H), 6.48 (d, *J* = 16.0 Hz, 1H), 4.84–4.66 (m, 3H), 4.18 (s, 1H), 4.01 (t, *J* = 6.4 Hz, 2H), 3.74 (s, 1H), 3.71 (s, 3H), 3.60–3.51 (m, 1H), 3.21 (s, 1H), 3.08–3.00 (m, 1H), 2.98–2.88 (m, 1H), 2.85–2.80 (m, 1H), 2.78–2.72 (m, 1H), 2.39 (s, 1H), 1.79–1.67 (m, 2H), 1.43 (s, 2H), 1.39–1.29 (m, 2H), 1.24 (s, 2H).^13^C NMR (125 MHz, DMSO) δ 167.40, 161.09, 144.84, 130.62, 126.89, 115.41, 115.29, 79.69, 71.24, 69.91, 68.18, 67.23, 59.58, 57.36, 52.43, 51.80, 29.00, 24.74, 23.96.HRMS (ESI) *m/z* [M + H]^+^: calcd for C_21_H_31_NO_7_: 410.2173, found 410.2170.

#### 5.3.10. Methyl(E*)-3-(4-(5-(3,4,5-trihydroxy-2-(hydroxymethyl)piperidin-1-yl)ethoxy)phenyl)acrylate (34)*

Pale brown solid; yield: 35%; m.p. 110–113 °C; purity: 95.5%. ^1^H NMR (500 MHz, DMSO) δ 7.65 (d, *J* = 8.6 Hz, 2H), 7.61 (d, *J* = 16.0 Hz, 1H), 6.97 (d, *J* = 8.6 Hz, 2H), 6.47 (d, *J* = 16.0 Hz, 1H), 4.99 (s, 4H), 4.01 (t, *J* = 6.2 Hz, 2H), 3.71 (s, 3H), 3.66 (s, 1H), 3.17 (s, 2H), 3.03 (s, 2H), 2.90 (s, 2H), 2.17 (d, *J* = 7.5 Hz, 1H), 1.74–1.69 (m, 2H), 1.46–1.38 (m, 4H), 1.32–1.27 (m, 2H).^13^C NMR (125 MHz, DMSO) δ 167.37, 161.10, 144.80, 132.59, 130.58, 126.92, 115.44, 115.30, 114.45, 79.71, 71.34, 69.93, 68.15, 67.17, 59.66, 57.42, 52.49, 51.72, 29.07, 27.21, 25.90, 24.98. HRMS (ESI) *m/z* [M + H]^+^: calcd for C_22_H_33_NO_7_: 424.2330, found424.2327.

#### 5.3.11. Ethyl(E)-3-(3-methoxy-4-(2-(3,4,5-trihydroxy-2-(hydroxymethyl)piperidin-1-yl)ethoxy)phenyl)acrylate (35)

Pale yellow solid; yield: 32%; m.p. 146–148 °C; purity: 91.2%. ^1^H NMR (500 MHz, DMSO) δ 7.58 (d, *J* = 15.9 Hz, 1H), 7.35 (s, 1H), 7.22 (d, *J* = 8.2 Hz, 1H), 7.01 (d, *J* = 8.3 Hz, 1H), 6.55 (d, *J* = 15.9 Hz, 1H), 4.78 (s, 3H), 4.21–4.15 (m, 2H), 4.15–4.05 (m, 2H), 3.80 (s, 3H), 3.61 (s, 1H), 3.29–3.13 (m, 2H), 3.12–3.02 (m, 2H), 2.96 (s, 2H), 2.86 (s, 1H), 2.14 (d, *J* = 42.0 Hz, 2H), 1.26 (t, *J* = 7.1 Hz, 3H).^13^C NMR (125 MHz, DMSO) δ 166.99, 149.57, 145.01, 123.36, 116.15, 113.10, 111.12, 77.58, 73.23, 69.24, 67.15, 65.81, 60.25, 57.49, 56.21, 51.19, 45.16, 14.72.HRMS (ESI) *m/z* [M + Na]^+^: calcd for C_20_H_29_NO_8_: 434.1785, found 434.1785.

#### 5.3.12. Ethyl(E)-3-(3-methoxy-4-(3-(3,4,5-trihydroxy-2-(hydroxymethyl)piperidin-1-yl)ethoxy)phenyl)acrylate (36)

Yellow oil; yield: 34%; purity: 90.5%.^1^H NMR (500 MHz, DMSO) δ 7.58 (d, *J* = 16.1 Hz, 1H), 7.35 (s, 1H), 7.22 (d, *J* = 7.9 Hz, 1H), 6.99 (d, *J* = 8.1 Hz, 1H), 6.55 (d, *J* = 15.9 Hz, 1H), 5.07 (d, *J* = 128.1 Hz, 3H), 4.23–4.10 (m, 2H), 4.04 (s, 2H), 3.81 (s, 3H), 3.76 (s, 1H), 3.68 (d, *J* = 12.7 Hz, 1H), 3.15 (d, *J* = 26.2 Hz, 2H), 3.05 (s, 3H), 1.95 (s, 2H), 1.23 (t, *J* = 7.1 Hz, 3H).^13^C NMR (125 MHz, DMSO) δ 166.98, 149.69, 144.98, 123.32, 116.20, 113.32, 111.25, 77.60, 73.25, 69.24, 67.13, 65.79, 60.30, 60.26, 56.23, 56.04, 51.20, 14.71, 14.54. HRMS (ESI) *m/z* [M + Na]^+^: calcd for C_21_H_31_NO_8_: 448.1942, found 448.1960.

#### 5.3.13. Ethyl(E)-3-(3-methoxy-4-(4-(3,4,5-trihydroxy-2-(hydroxymethyl)piperidin-1-yl)ethoxy)phenyl)acrylate (37)

Yellow oil; yield: 35%; purity: 91.9%. ^1^H NMR (500 MHz, DMSO) δ 7.58 (d, *J* = 15.9 Hz, 1H), 7.35 (d, *J* = 1.4 Hz, 1H), 7.22 (d, *J* = 8.4, 1H), 6.98 (d, *J* = 8.5 Hz, 1H), 6.54 (d, *J* = 15.9 Hz, 1H), 4.76 (s, br, 3H), 4.22–4.14 (m, 2H), 4.03 (s, 2H), 3.81 (s, 3H), 3.76 (s, 1H), 3.75 (s, 1H), 3.62 (s, 1H), 3.26 (s, 2H), 3.10 (s, 1H), 3.03–2.83 (m, 4H), 1.71–1.66 (m, 2H), 1.56 (s, 2H), 1.26 (t, *J* = 7.1 Hz, 3H).^13^C NMR (125 MHz, DMSO) δ 167.00, 149.62, 145.06, 123.36, 116.04, 113.05, 111.09, 77.65, 73.30, 68.54, 66.75, 65.75, 60.29, 60.24, 56.15, 55.94, 51.99, 26.93, 14.72, 14.55. HRMS (ESI) *m/z* [M + Na]^+^: calcd for C_22_H_33_NO_8_: 440.2279, found 440.2283.

#### 5.3.14. Ethyl(E)-3-(3-methoxy-4-(5-(3,4,5-trihydroxy-2-(hydroxymethyl)piperidin-1-yl)ethoxy)phenyl)acrylate (38)

Yellow oil; yield: 32%; purity: 90.6%. ^1^H NMR (500 MHz, DMSO) δ 7.61 (d, *J* = 15.9, 1H), 7.36 (s, 1H), 7.24 (s, 1H), 7.03 (d, *J* = 8.2 Hz, 1H), 6.56 (d, *J* = 15.9, 1H), 4.78 (s, 4H), 4.21–4.15 (m, 2H), 4.15–4.11 (m, 2H), 3.81 (s, 3H), 3.77–3.70 (m, 2H), 3.71 (s, 2H), 3.67 (s, 1H), 3.51 (s, 2H), 3.28 (s, 3H), 3.16–3.05 (m, 2H), 3.00 (s, 2H), 2.18 (s, 2H), 1.24 (t, *J* = 7.1 Hz, 3H).13C NMR (125 MHz, DMSO) δ 167.00, 149.57, 145.02, 123.34, 116.07, 112.99, 111.03, 77.59, 73.35, 68.52, 66.17, 65.59, 60.29, 60.26, 56.16, 55.94, 51.28.60, 23.48, 14.72, 14.55.HRMS (ESI) *m/z* [M + Na]^+^: calcd for C_23_H_35_NO_8_: 476.2255, found476.2256.

#### 5.3.15. Ethyl(E)-3-(3-methoxy-4-(6-(3,4,5-trihydroxy-2-(hydroxymethyl)piperidin-1-yl)ethoxy)phenyl)acrylate (39)

Yellow oil; yield: 35%; purity: 92.1%. ^1^H NMR (500 MHz, DMSO) δ 7.57 (d, *J* = 15.9 Hz, 1H), 7.33 (d, *J* = 1.7 Hz, 1H), 7.20 (d, *J* = 8.3, 1H), 6.95 (d, *J* = 8.4 Hz, 1H), 6.52 (d, *J* = 15.9 Hz, 1H), 4.72 (s, 3H), 4.22–4.15 (m, 2H), 3.97 (t, *J* = 6.5 Hz, 2H), 3.81 (s, 3H), 3.74 (d, *J* = 10.8, 9.2 Hz, 1H), 3.58 (dd, *J* = 11.5, 3.0 Hz, 1H), 3.28–3.20 (m, 1H), 3.18 (s, 1H), 3.08 (t, *J* = 9.1 Hz, 1H), 2.94 (t, *J* = 8.8 Hz, 1H), 2.83 (dd, *J* = 11.0, 4.7 Hz, 1H), 2.80–2.72 (m, 1H), 2.46–2.37 (m, 1H), 2.04–1.93 (m, 2H), 1.77–1.65 (m, 2H), 1.47–1.34 (m, 4H), 1.29–1.20 (m, 5H).^13^C NMR (125 MHz, DMSO) δ 166.96, 150.89, 149.65, 144.99, 127.25, 123.29, 116.04, 113.05, 111.19, 79.57, 71.17, 69.77, 68.68, 67.07, 60.21, 59.40, 57.25, 56.17, 52.50, 49.06, 29.13, 27.17, 25.91, 24.86, 14.70.HRMS (ESI) *m/z* [M + H]^+^: calcd for C_24_H_37_NO_8_: 468.2592, found 468.2589.

#### 5.3.16. (E)-3-phenyl-1-(2-(2-(3,4,5-trihydroxy-2-(hydroxymethyl)piperidin-1-yl)ethoxy)phenyl)prop-2-en-1-one (40)

Yellow oil; yield: 31%; purity: 95.1%. ^1^H NMR (500 MHz, DMSO) δ 7.77–7.71 (m, 2H), 7.54 (s, 1H), 7.53 (s, 2H), 7.52 (s, 1H), 7.45 (d, *J* = 1.9 Hz, 1H), 7.45–7.43(m, 2H), 7.23 (d, *J* = 8.2 Hz, 1H), 7.09–7.03 (m, 1H), 4.70 (s, br, 3H), 4.30–4.17 (m, 3H), 3.80–3.75 (m, 1H), 3.53 (d, *J* = 9.6 Hz, 1H), 3.22 (s, 2H), 3.04–2.98 (m, 1H), 2.93 (s, 2H), 2.13 (s, 1H), 2.09 (s, 2H).^13^C NMR (125 MHz, DMSO) δ 192.24, 157.83, 142.52, 135.11, 133.68, 130.84, 130.20, 129.47, 129.37, 128.95, 127.46, 121.10, 113.86, 79.53, 77.60, 73.25, 70.89, 69.24, 67.40, 65.80, 57.52, 51.50, 45.21. HRMS (ESI) *m/z* [M + Na]^+^: calcd for C_23_H_27_NO_6_: 436.1731, found436.1744.

#### 5.3.17. (E)-3-Phenyl-1-(2-(3-(3,4,5-trihydroxy-2-(hydroxymethyl)piperidin-1-yl)ethoxy)phenyl)prop-2-en-1-one (41)

Yellow oil; yield: 34%; purity: 92.0%. ^1^H NMR (500 MHz, DMSO) δ 7.77–7.70 (m, 2H), 7.54–7.53 (m, 1H), 7.53–7.51 (m, 2H), 7.51 (s, 1H), 7.45 (d, *J* = 2.1 Hz, 1H), 7.44 (s, 1H), 7.32–7.24 (m, 1H), 7.20–7.16 (m, 1H), 7.08–7.03 (m, 1H), 4.66 (s, 3H), 4.14–4.10 (m, 2H), 4.09 (s, 1H), 3.68 (d, *J* = 11.4 Hz, 1H), 3.49 (d, *J* = 12.0 Hz, 1H), 3.19 (s, 1H), 3.07–2.97 (m, 1H), 2.95–2.85 (m, 2H), 2.80–2.71 (m, 1H), 2.49–2.43 (m, 1H), 2.09 (s, 2H), 1.95–1.85 (m, 2H). ^13 ^C NMR (125 MHz, DMSO) δ 192.34, 157.84, 142.47, 135.11, 133.68, 130.85, 130.20, 129.46, 129.31, 128.87, 128.44, 127.58, 121.00, 113.65, 79.58, 71.24, 69.82, 67.57, 67.14, 59.61, 57.48, 49.20, 31.13, 25.42.HRMS (ESI) *m/z* [M + Na]^+^: calcd for C_24_H_29_NO_6_: 450.1887, found450.1898.

#### 5.3.18. (E)-3-phenyl-1-(2-(4-(3,4,5-trihydroxy-2-(hydroxymethyl)piperidin-1-yl)ethoxy)phenyl)prop-2-en-1-one (42)

Yellow oil; yield: 34%; purity: 95.8%.^1^H NMR (500 MHz, DMSO) δ 7.74–7.68 (m, 2H), 7.56–7.49 (m, 4H), 7.48–7.42 (m, 3H), 7.19 (d, *J* = 8.6 Hz, 1H), 7.07–7.03 (m, 1H), 4.77–4.57 (m, 3H), 4.12 (t, *J* = 6.3 Hz, 3H), 3.67 (d, *J* = 11.4 Hz, 1H), 3.56–3.48 (m, 1H), 3.18 (s, 1H), 3.09–2.99 (m, 1H), 2.94–2.87 (m, 1H), 2.79–2.68 (m, 2H), 2.33–2.25 (m, 1H), 1.90–1.81 (m, 2H), 1.75–1.59 (m, 2H), 1.57–1.42 (m, 2H). ^13 ^C NMR (125 MHz, DMSO) δ 192.39, 157.84, 142.43, 135.08, 133.68, 130.86, 130.19, 129.48, 129.30, 128.82, 127.58, 120.95, 113.66, 79.64, 71.22, 69.84, 68.62, 67.29, 59.47, 57.09, 51.91, 49.07, 27.15, 21.73.HRMS (ESI) *m/z* [M + H]^+^: calcd for C_25_H_31_NO_6_: 442.2224, found 442.2222.

#### 5.3.19. (E)-3-phenyl-1-(2-(5-(3,4,5-trihydroxy-2-(hydroxymethyl)piperidin-1-yl)ethoxy)phenyl)prop-2-en-1-one (43)

Yellow oil; yield: 37%; purity: 95.3%. ^1^H NMR (500 MHz, DMSO) δ 7.75–7.69 (m, 2H), 7.55–7.49 (m, 4H), 7.47–7.42 (m, 3H), 7.18 (d, *J* = 8.6 Hz, 1H), 7.07–7.03 (m, 1H), 4.68 (s, 3H), 4.08 (t, *J* = 6.2 Hz, 2H), 3.64 (d, *J* = 11.0 Hz, 1H), 3.56–3.48 (m, 1H), 3.25–3.15 (m, 2H), 3.04 (t, *J* = 8.8 Hz, 1H), 2.91 (t, *J* = 8.9 Hz, 1H), 2.77–2.68 (m, 1H), 2.64–2.54 (m, 1H), 2.31–2.18 (m, 1H), 1.99–1.83 (m, 2H), 1.70 (s, 2H), 1.30 (s, 4H).^13^C NMR (125 MHz, DMSO) δ 192.40, 157.89, 142.28, 137.58, 135.11, 133.70, 130.86, 130.18, 129.44, 129.32, 129.00, 128.81, 127.68, 120.96, 113.59, 79.63, 71.20, 69.82, 68.71, 66.99, 59.40, 57.30, 52.31, 29.19, 24.71, 24.07.HRMS (ESI) *m/z* [M + H]^+^: calcd for C_26_H_33_NO_6_: 456.2381, found456.2376.

#### 5.3.20. (E)-3-phenyl-1-(2-(6-(3,4,5-trihydroxy-2-(hydroxymethyl)piperidin-1-yl)ethoxy)phenyl)prop-2-en-1-one (44)

Yellow oil; yield: 35%; purity: 94.7%. ^1^H NMR (500 MHz, DMSO) δ 7.76–7.68 (m, 2H), 7.56–7.49 (m, 4H), 7.46–7.43 (m, 3H), 7.18 (d, *J* = 8.2 Hz, 1H), 7.08–7.02 (m, 1H), 4.72 (s, 3H), 4.09 (t, *J* = 6.0 Hz, 2H), 3.66 (d, *J* = 11.3 Hz, 1H), 3.54 (s, 1H), 3.22 (s, 1H), 3.07 (s, 1H), 2.94 (s, 1H), 2.75 (s, 1H), 2.65 (s, 1H), 2.32 (s, 1H), 1.95 (s, 2H), 1.76–1.63 (m, 2H), 1.42–1.30 (m, 2H), 1.26–1.16 (m, 2H), 1.15–1.04 (m, 2H). ^13 ^C NMR (125 MHz, DMSO) δ 192.43, 167.48, 157.88, 142.26, 135.10, 133.85, 133.71, 131.59, 130.88, 130.18, 129.44, 129.30, 128.81, 127.72, 120.97, 113.53, 89.65, 68.68, 66.90, 56.14, 52.46, 40.97, 31.14, 29.32, 27.25, 26.18, 25.03.HRMS (ESI) *m/z* [M + H]^+^: calcd for C_27_H_35_NO_6_: 470.2537, found470.2524.

### 5.4. *In vitro* assay of α-glucosidase inhibitory activity

The method we used was reported before in our team^23^, α-glucosidase inhibitory activity was measured by using 0.1 mM phosphate buffer (pH 6.8) at 37 °C. The α-glucosidase enzyme (EC 3.2.1.20, 1 U/ml, 10 µL) in phosphate buffer was incubated with various concentrations of tested compounds (dissolved in 1% DMSO) at 37 °C for 20 min, then PNPG (10 mM, 20 µL) was added to the mixture as substrate. Finally, the absorbance was measured at 405 nm by using a spectrophotometer. The sample solution was replaced by DMSO as the control. Acarbose and 1-DNJ were used as standard drugs. The inhibition has been obtained using the formula:

$$\mathrm{Inhibition}\;(\%)=(\Delta A_{\mathrm{control}}-\Delta A_{\mathrm{sample}})/\Delta A_{\mathrm{control}}\times 100.{\text{ The IC}}_{50}\text{ fitted with SPSS}.$$

### 5.5. Kinetic study

The kinetic analysis was carried out to ensure inhibition mode of the three most active compounds **43**, **40**, and **34**. The 10 µL of enzyme solution (1 U/mL) was incubated with different concentrations of compound **43** (0.00 mM,0.012 mM, 0.02 mM, 0.04 mM), **40** (0.00 mM, 0.06 mM, 0.10 mM, 0.20 mM)and **34** (0.00 mM, 0.30 mM, 0.50 mM, 1.00 mM) for 20 min at 37 °C, then added different concentrations of substrate (1 mM, 0.9 mM, 0.75 mM, 0.6 mM, 0.45 mM, 0.3 mM, 0.25 mM), and change in absorbance was measured for 20 min at 405 nm by using spectrophotometer.

### 5.6. Molecular docking

Since the X-ray crystallographic structure of *Saccharomyces cerevisiae* α-glucosidase we used in the experiments has not been reported yet, the 3 D structural modelling of α-glucosidase was conducted with SWISS-MODEL^22^. The sequence in the FASTA format of α-glucosidase was download from NCBI. Isomaltase from *Saccharomyces cerevisiae*(PDB code 3AJ7) shows high sequence similarity (72.51%) with α-glucosidase, which structure was selected as the template for homology modelling, and the quality of the obtained homology model was verified using PROCHECK^25^. The 3 D structures of acarbose and synthesised compounds were built by ChemBioDraw Ultra and ChemBio3D Ultra software. The AutoDock Tool 1.5.6 package was employed to generate the docking input files. Docking studies were performed using Autodock Vina^21^. The centre of the grid box^26^ was placed at centre_x = 12.5825, centre_y = −7.8955, centre_z = 12.5190 with dimensions size_x = 40, size_y = 40, size_z = 40. The best-scoring poses as judged by the Vina docking score were chosen and visually analysed using PyMOL 1.8.0 software (http://www.pymol.org/).
